# RTX-KG2: a system for building a semantically standardized knowledge graph for translational biomedicine

**DOI:** 10.1186/s12859-022-04932-3

**Published:** 2022-09-29

**Authors:** E. C. Wood, Amy K. Glen, Lindsey G. Kvarfordt, Finn Womack, Liliana Acevedo, Timothy S. Yoon, Chunyu Ma, Veronica Flores, Meghamala Sinha, Yodsawalai Chodpathumwan, Arash Termehchy, Jared C. Roach, Luis Mendoza, Andrew S. Hoffman, Eric W. Deutsch, David Koslicki, Stephen A. Ramsey

**Affiliations:** 1 School of Electrical Engineering and Computer Science, Oregon State University, Corvallis, OR USA; 2 Computer Science and Engineering, Penn State University, State College, PA USA; 3 Huck Institutes of the Life Sciences, Penn State University, State College, PA USA; 4 Institute for Systems Biology, Seattle, WA USA; 5 Interdisciplinary Hub for Digitalization and Society, Radboud University, Nijmegen, The Netherlands; 6 Department of Biology, Penn State University, State College, PA USA; 7 King Mongkut’s University of Technology North Bangkok, Bangkok, Thailand; 8 Department of Biomedical Sciences, Oregon State University, Corvallis, OR USA

**Keywords:** Knowledge graph, Biomedical knowledge integration, Semantic normalization

## Abstract

****Background**:**

Biomedical translational science is increasingly using computational reasoning on repositories of structured knowledge (such as UMLS, SemMedDB, ChEMBL, Reactome, DrugBank, and SMPDB in order to facilitate discovery of new therapeutic targets and modalities. The NCATS Biomedical Data Translator project is working to federate autonomous reasoning agents and knowledge providers within a distributed system for answering translational questions. Within that project and the broader field, there is a need for a framework that can efficiently and reproducibly build an integrated, standards-compliant, and comprehensive biomedical knowledge graph that can be downloaded in standard serialized form or queried via a public application programming interface (API).

****Results**:**

To create a *knowledge provider* system within the Translator project, we have developed RTX-KG2, an open-source software system for building—and hosting a web API for querying—a biomedical knowledge graph that uses an Extract-Transform-Load approach to integrate 70 knowledge sources (including the aforementioned core six sources) into a knowledge graph with provenance information including (where available) citations. The semantic layer and schema for RTX-KG2 follow the standard Biolink model to maximize interoperability. RTX-KG2 is currently being used by multiple Translator reasoning agents, both in its downloadable form and via its SmartAPI-registered interface. Serializations of RTX-KG2 are available for download in both the pre-canonicalized form and in canonicalized form (in which synonyms are merged). The current canonicalized version (KG2.7.3) of RTX-KG2 contains 6.4M nodes and 39.3M edges with a hierarchy of 77 relationship types from Biolink.

****Conclusion**:**

RTX-KG2 is the first knowledge graph that integrates UMLS, SemMedDB, ChEMBL, DrugBank, Reactome, SMPDB, and 64 additional knowledge sources within a knowledge graph that conforms to the Biolink standard for its semantic layer and schema. RTX-KG2 is publicly available for querying via its API at arax.rtx.ai/api/rtxkg2/v1.2/openapi.json. The code to build RTX-KG2 is publicly available at github:RTXteam/RTX-KG2.

**Supplementary Information:**

The online version contains supplementary material available at 10.1186/s12859-022-04932-3.

## Background

In translational biomedicine, there is a longstanding need to integrate structured knowledge as a substrate for computational reasoning [1], such as for drug repositioning or finding new therapies for monogenic disorders. Efforts to define a *lingua franca* for a computable and comprehensive biomedical knowledge graph have seen a pivot from controlled vocabularies [2–6] (and their integration in the Unified Medical Language System (UMLS) Metathesaurus [7]) to ontologies in standardized computable representations [8–10]. The World Wide Web has fueled the development of online knowledge-bases updated by literature curation teams, such as KEGG [11], PubChem [12], DrugBank [13], ChEMBL [14], the UniProt Knowledgebase (UniProtKB) [15], the Small Molecule Pathway Database (SMPDB) [16, 17], and Reactome [18]. At the same time, advances in natural language processing (NLP) [19–24] have enabled systematic extraction of structured knowledge from the biomedical literature, such as the Semantic MEDLINE [25] Database (SemMedDB) [26]. Community-driven ontology development [27–31], literature curation, and the use of NLP together have driven growth of structured biomedical knowledge-bases, albeit in forms that are not semantically interoperable due to the use of different systems of concept identifiers, semantic types, and relationship types [32].

There have been numerous efforts to address the lack of semantic interoperability of structured biomedical knowledge, particularly in knowledge representation [33]. BIOZON [34], BioGraphDB [35], Hetionet [36], SPOKE [37, 38], EpiGraphDB [39], and DRKG [40] used standard sets of identifier types; and Bio2RDF [41], KaBOB [42], and HKGB [43] used ontologies [30, 31] for knowledge linking. ROBOKOP [44, 45], BioThings [46], and mediKanren [47, 48] use concept and relationship types from the recently-developed Biolink model [49–51]. Biolink is a high-level ontology that provides mappings of semantic types and relation types to other ontologies. Biolink advanced the field by (i) providing mappings of semantic types and relation types to other ontologies; (ii) standardizing and ranking preferred identifier types for various biological entities; and (iii) providing hierarchies of relation types and concept types needed to provide a semantic layer for biological knowledge graphs. In 2016, the National Center for Advancing Translational Sciences (NCATS) launched the Biomedical Data Translator project [52], a multi-institution effort to develop a distributed computational reasoning and knowledge exploration system for translational science. After a feasibility assessment phase in 2017–2020, the project began construction of the Translator system’s components such as reasoning agents, knowledge providers, and central controller system in 2020. The RTX-KG2 system is a registered knowledge provider within Translator. To ensure that Translator’s various systems can interoperate, Biolink has been adapted as the semantic layer for concepts and relations for knowledge representation within the Translator project.

Because biomedical knowledge-bases collectively use various semantically overlapping controlled vocabularies for concept types like diseases, drugs, phenotypes, and pathways, integrating knowledge into a graph entails grappling with the problem of multiple identifiers for a single concept; for example, the concept “paracetamol” has many identifiers, such as UMLS:C0000970, DRUGBANK:DB00316, CHEBI:46195, and CHEMBL112. While most biomedical knowledge graph efforts map concepts to canonical identifiers from semantic type-specific controlled vocabularies during initial graph construction, Monarch [53–55] constructed a linked graph of concept identifiers and then used clique detection to identify identical concepts before selecting a representative canonical identifier (a step that is called graph “canonicalization” [56]) for each clique. To date, biomedical knowledge graphs of which we are aware (with the exception of Bio2RDF [41]) are either canonicalized or standardized on a single identifier type for each semantic type, rather than providing *both* canonicalized and pre-canonicalized graphs; the latter form is important in order to support users that wish to apply their own canonicalization algorithm.

Previous efforts to develop integrated biomedical knowledge systems have used various database types, architectural patterns, and automation frameworks. For persistence, knowledge systems have used relational databases [34], distributed graph databases [33, 57], multimodal NoSQL databases [35, 57], RDF triple-stores [41, 42, 58], document-oriented databases [32, 46, 54], and—with increasing frequency [36, 37, 39, 44, 54]—the open-source graph database Neo4j (github:neo4j/neo4j). Knowledge systems have also differed in terms of the ingestion method used in their construction; many systems [32, 35, 41, 42, 54] utilized an extract-transform-load (ETL) approach, whereas others [44, 46, 59] used API endpoints to query upstream knowledge sources; one [39] blended both ETL and API approaches for knowledge graph construction. For automation, previous efforts have used general-purpose scripting languages  [36, 37, 41, 42, 44, 59, 60], batch frameworks [32], rule-based build frameworks [33, 35, 61], semantic web-compliant build frameworks such as PheKnowLator [62], and parallel-capable systems such as Snakemake [63]. While previous efforts have resulted in biomedical knowledge graphs incorporating (individually) UMLS, SemMedDB, multiple major drug knowledge bases (such as ChEMBL and DrugBank), a standards-compliant semantic layer, and a parallel build system, so far as we are aware, none have incorporated all of these features in a single system providing both canonicalized and pre-canonicalized knowledege graphs.

### Introduction

We have developed RTX-KG2, an open-source biomedical knowledge graph representing biomedical concepts and their relationships. RTX-KG2 integrates 70 sources including the major sources UMLS, SemMedDB, ChEMBL, DrugBank, SMPDB, Reactome, KEGG, and UniProtKB using a modular build system leveraging the parallel-capable workflow framework, Snakemake. The semantic layer for RTX-KG2 is based on the standards-based Biolink model and it is provided in two stages, a pre-canonicalized graph version (RTX-KG2pre, in which semantically duplicated concepts with distinct identifiers are distinct nodes) and a canonicalized version (RTX-KG2c) in which equivalent concepts described using different identifier systems are identified as a single node. These key design choices reflect the goals of (i) supporting interoperability and composability with other biomedical knowledge-bases in the Translator system and (ii) providing a comprehensive knowledge graph with a standards-based semantic layer that is amenable to computational reasoning. Both RTX-KG2pre and RTX-KG2c are directed multigraphs with node and edge annotations that follow the Biolink model. The software repository for RTX-KG2, including all code to build the database, is publicly available at the github:RTXteam/RTX-KG2 GitHub project. Users can access RTX-KG2 content via any of three channels: (i) a single-file download version of the canonicalized RTX-KG2 knowledge graph (KG2c) (or, if needed, the pre-canonicalized RTX-KG2pre knowledge graph); (ii) a publicly accessible, SmartAPI [64]-registered API for querying RTX-KG2; and (iii) a web browser interface for querying RTX-KG2. RTX-KG2 uses an ETL approach for knowledge graph construction and it automates builds using Snakemake; together, these enable efficient knowledge graph construction. RTX-KG2 is a built-in knowledge database for ARAX (Autonomous Relay Agent X) [65], a Web-based computational biomedical reasoning system that our team is also developing for answering translational science questions such as questions related to drug repositioning, identifying new therapeutic targets, and understanding drug mechanisms-of-action. We are developing RTX-KG2 and ARAX as a part of the NCATS Translator project. Here, we enumerate the knowledge sources that are incorporated into RTX-KG2 (in the "Sources and their file formats" section; outline the processes for building RTX-KG2pre from its upstream knowledge sources (in the "Building RTX-KG2pre from upstream sources" section) and for building the canonicalized RTX-KG2c (in the "Building RTX-KG2c, the canonicalized version of RTX-KG2" section); describe the schema for RTX-KG2 (in the "RTX-KG2 schema and RTX-KG2pre Biolink compliance" section); describe the RTX-KG2 build system software (in the "RTX-KG2 build system and software" section); provide statistics about the size and semantic breadth of RTX-KG2 (in the "RTX-KG2 content and statistics" section); and discuss how it is being used for translational reasoning as well as in conjunction with the ARAX system (in the "Utility and discussion" section).

## Construction and content

**Fig. 1:**
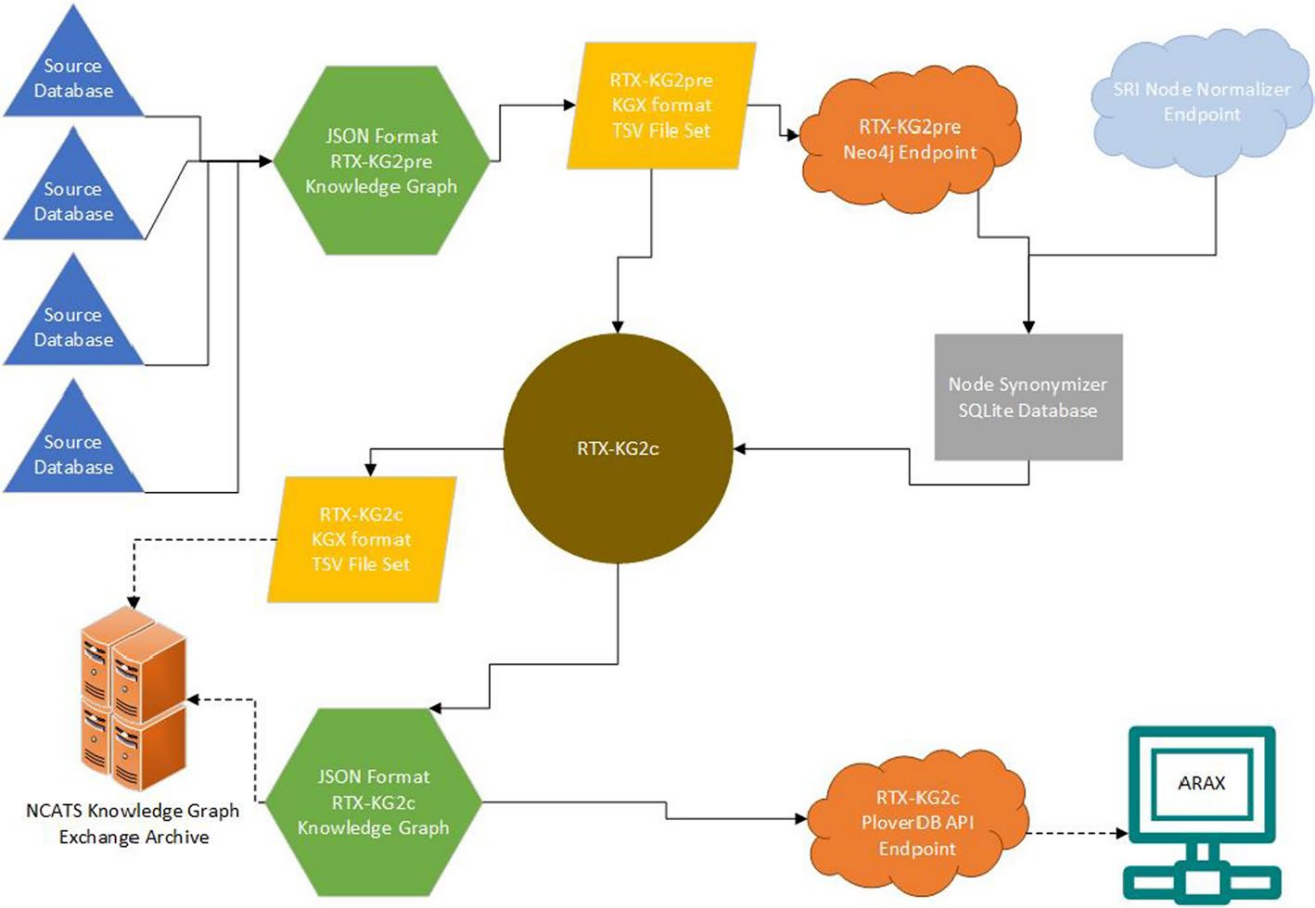
Overall Workflow of RTX-KG2. Blue triangle: individual external source; light blue cloud: external API endpoint; yellow parallelogram: tab-separated value (TSV) file-set; green hexagon: JavaScript Object Notation (JSON) File; orange cloud: API endpoint output; grey rectangle: SQLite [66] database; brown circle: abstract object-model representation of KG2c; turquoise computer: user/client computer; orange server: Translator knowledge graph exchange (KGE) server

In this section, we describe how RTX-KG2 is constructed; provide an overview of its graph database schema; and summarize its content in terms of sources, semantic breadth, and size. The overall build process, along with the various outputs of RTX-KG2, is depicted in Fig. 1.

Broadly speaking, the RTX-KG2 build system does four things: it (i) loads information from source databases (blue triangles in Fig. 1) via the World Wide Web as described in the "Sources and their file formats" section; (ii) integrates the knowledge into a precursor knowledge graph called RTX-KG2pre (upper green hexagon in Fig. 1) and hosts it in a Neo4j database (upper orange cloud in Fig. 1) as described in the "Building RTX-KG2pre from upstream sources" section; (iii) coalesces equivalent concept nodes into a canonicalized knowledge graph called RTX-KG2c (brown circle in Fig. 1) as described in the "Building RTX-KG2c, the canonicalized version of RTX-KG2" section, with a schema that is described in the "RTX-KG2 schema and RTX-KG2pre Biolink compliance" section; and (iv) provides various knowledge graph artifacts and services as described in the "RTX-KG2 content and statistics" section. We provide technical details of the RTX-KG2 build system in the "RTX-KG2 build system and software" section.

### Sources and their file formats

RTX-KG2 integrates 70 sources (Table 1), 50 of them via a *resource description framework (RDF)-based* ingestion method and 20 of them via a *direct-to-JSON* ingestion method.

**Table 1:** RTX-KG2 integrates 70 knowledge sources into a single graph. Each row represents a server site from which sources were downloaded.

Name	#	Description	Format	Method
Biolink [49, 50]	1	Biolink model (semantic layer)	TTL	RBM
ChEMBL [14, 69]	1	EMBL chemogenomic database	SQL	D2J
DGIdb [70]	1	Drug gene interaction database	TSV	D2J
DisGeNET [71]	1	Disease-gene associations	TSV	D2J
DrugBank [13]	1	Pharmaceutical knowledge base	XML	D2J
DrugCentral [72]	1	Online drug compendium	SQL	D2J
Ensembl Gene [73]	1	Ensembl human gene annotations	JSON	D2J
EFO [74]	1	Experimental Factor ontology	OWL	RBM
GO [75, 76]	1	Gene ontology annotations	TSV	D2J
HMDB [77–80]	1	Human metabolite database	XML	D2J
IntAct [81, 82]	1	IntAct molecular interaction database	TSV	D2J
Jensen Lab Diseases [83]	1	Gene to diseases dataset	TSV	D2J
KEGG [11, 84, 85]	1	Kyoto encyclopedia of genes and genomes	API	D2J
miRBase [86–90]	1	MicroRNAs dataset	DAT	D2J
NCBI Gene [91]	1	NCBI human gene annotations	TSV	D2J
OBO Foundry	21	OBO foundry ontologies (Additional file 1: Table S1)	OWL	RBM
Orphanet [92]	1	Orphanet rare disease ontology	OWL	RBM
PathBank [93–95]	1	Wishart lab pathway databases	XML	D2J
Reactome [96]	1	Pathway database	SQL	D2J
SemMedDB [26]	1	Semantic MEDLINE database	SQL	D2J
SMPDB [16, 17]	1	Small molecule pathway database	CSV	D2J
UMLS [97]	26	Unified medical language system (Table 7)	TTL	RBM
UniChem [98]	1	EBI small molecule cross-refs	TSV	D2J
UniProtKB [15]	1	UniProt knowledge base	DAT	D2J
Total	70			

Columns as follows: *Name,* the short name(s) of the knowledge sources obtained or the distribution name in the cases of UMLS and OBO Foundry; *#*, the number of individual sources or ontologies obtained from that server; *Format*, the file format used for ingestion (see below); *Method*, the ingestion method used for the source, either D2J for direct-to-JSON or RBM for the RDF-based method. File format codes: CSV, comma-separated value; DAT, SWISS-PROT-like DAT format; JSON, JavaScript object notation; OWL, OWL in RDF/XML [67] syntax; RRF, UMLS Rich Release Format [68]; SQL, structured query language (SQL) dump; TSV, tab-separated value; XML, extensible markup language. Other abbreviations: NCBI, National Center for Biotechnology Information; EMBL, European Molecular Biology Laboratory

Sources are loaded in a specific order controlled by a configuration file, with precedence applying to the assignment of Biolink categories to nodes.

#### RDF-based sources

Of the 50 RDF-based sources, the system ingests 27 in Terse RDF Triple Language (TTL [99]) format and 23 as OWL ontologies in RDF/XML format [67] (which we abbreviate here as “OWL”). Of the 27 TTL sources, 26 are from the UMLS, obtained as described in "umls2rdf and owltools" section; the remaining source is a TTL representation of the Biolink model, which defines the semantic layer for RTX-KG2, including hierarchies of concept types and relation types (see the "RTX-KG2 content and statistics" section). In addition to concept type and relation type hierarchies, the Biolink model provides equivalence mappings of the Biolink types to classes in other high-level ontologies (such as biolink:Gene being equivalent to SIO:010035) and of the Biolink concept types to prioritized lists of identifier types for the concept type^1^. Each knowledge source’s concepts are assigned Biolink concept semantic types—which are called “categories” in the Biolink model—and relationships are assigned Biolink relationship types at the time that the source is ingested. All but two of the 23 OWL-format sources are ontologies from the OBO Foundry [31]; the remaining two OWL-format sources are the Experimental Factor Ontology (EFO) [74] and Orphanet Rare Disease Ontology [100].

#### Direct-to-JSON sources

With the direct-to-JSON method, sources are ingested in seven different file formats (in "Sources and their file formats" section). One source, KEGG, is queried via an API rather than using a flat file download, due to the lack of a download option for users that do not have a commercial license. For the 20 direct-to-JSON sources, the RTX-KG2 system has one ETL module for each source, with each module producing a source-specific JSON file in the RTX-KG2 JSON schema (see the "RTX-KG2 schema and RTX-KG2pre Biolink compliance" section) (in contrast, for the 50 RDF-based sources, the system has a single ETL module for ingesting all sources together). The RDF-based method merges all of the OWL and TTL sources (class-based), without flattening the ontologies (i.e., preserving rdfs:subClassOf relationships) and generates a single JSON file. The hybrid design of RTX-KG2 balances the benefits of modularity (where it is feasible in the direct-to-JSON method) with the need for a monolithic ingestion module for ontologies where inter-ontology axioms are needed for determining semantic types at the ETL stage [101].

### Building RTX-KG2c, the canonicalized version of RTX-KG2

Because the various ontologies that RTX-KG2pre ingests often represent the same concept using multiple different identifiers, some of the nodes in RTX-KG2pre represent equivalent concepts. For example, *Parkinson’s disease* is represented by several nodes in RTX-KG2pre, such as the nodes with identifiers MONDO:0005180, DOID:14330, EFO:0002508, and MESH:D010300, many of which are connected in RTX-KG2pre with relationships of type biolink:same_as or non-transitive generalizations of that relationship type. In our work on RTX-KG2pre, we found that coalescing nodes for semantically equivalent concepts into single nodes facilitates reasoning by reducing the complexity of graph paths that represent answers for common translational questions. Thus, to enhance the utility of RTX-KG2 for translational reasoning, we created a version of RTX-KG2 called RTX-KG2canonicalized (abbreviated in this work as RTX-KG2c) in which semantically equivalent nodes are coalesced to a single concept node. In brief, building RTX-KG2c from RTX-KG2pre is carried out in five steps:

1. RTX-KG2pre nodes and edges are loaded from the RTX-KG2pre TSV files;
2. the set of nodes is partitioned into disjoint subsets of equivalent nodes;
3. from each group of equivalent nodes, a canonical node identifier is chosen, added to RTX-KG2c, and annotated with the identifiers of its synonymous nodes (along with other information merged from the synonymous nodes);
4. edges from RTX-KG2pre are remapped to refer only to the canonical node identifiers; and
5. edges with the same subject, object, and Biolink predicate are merged.

For Steps 2–3, the RTX-KG2 build system uses the ARAX [65] system’s *Node Synonymizer* service (see in "ARAX Node Synonymizer" section for details). The RTX-KG2c graph is serialized in JSON format (see the "RTX-KG2 schema and RTX-KG2pre Biolink compliance" section), archived in a GitHub large file storage (LFS) repository (see Availability of data and materials), and imported into a custom-built, open-source, in-memory graph database, PloverDB (github:RTXteam/PloverDB). The build process for RTX-KG2c is Python-based and has comparable hardware requirements to the RTX-KG2pre build process (see the "Requirements" section). Formally, the approach used in building RTX-KG2c is concept-oriented as opposed to the realist methodology underlying OBO Foundry ontologies [102].

### RTX-KG2 schema and RTX-KG2pre Biolink compliance

**Fig. 2:**
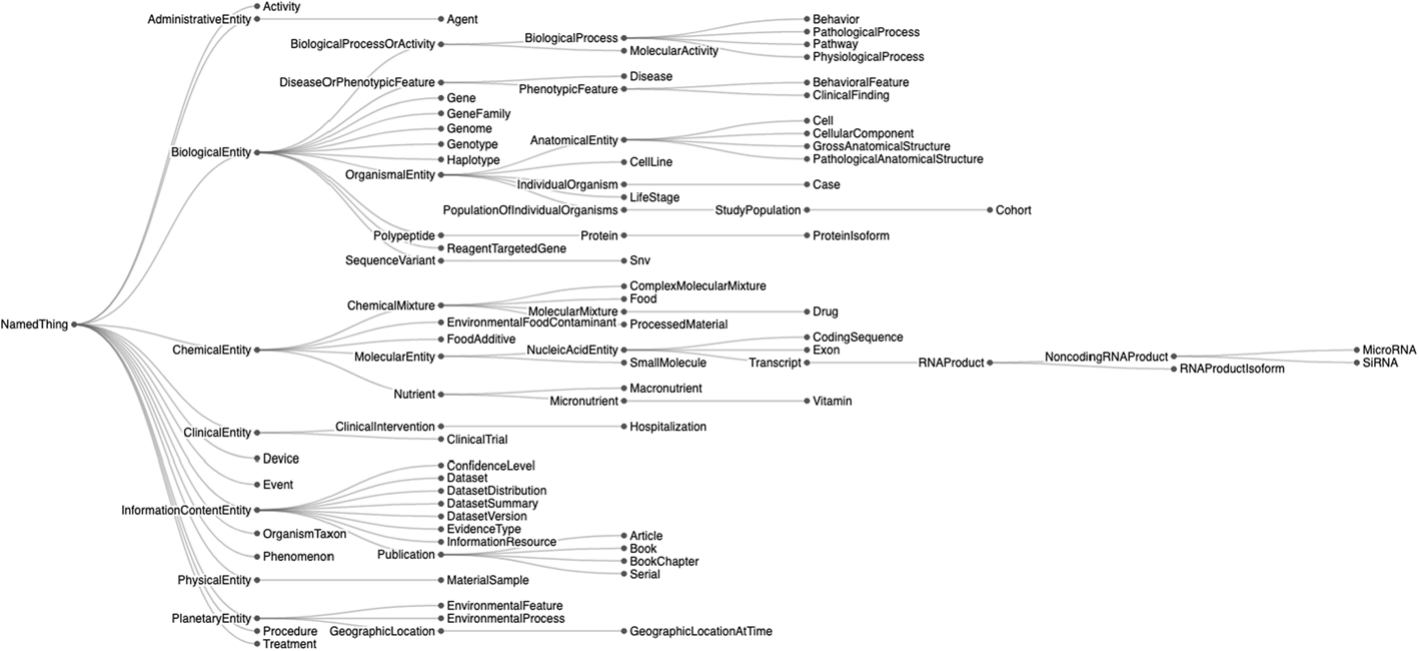
Node concept types in RTX-KG2.7.3 are based on the Biolink model version 2.1.0 [49, 50]

**Fig. 3:**
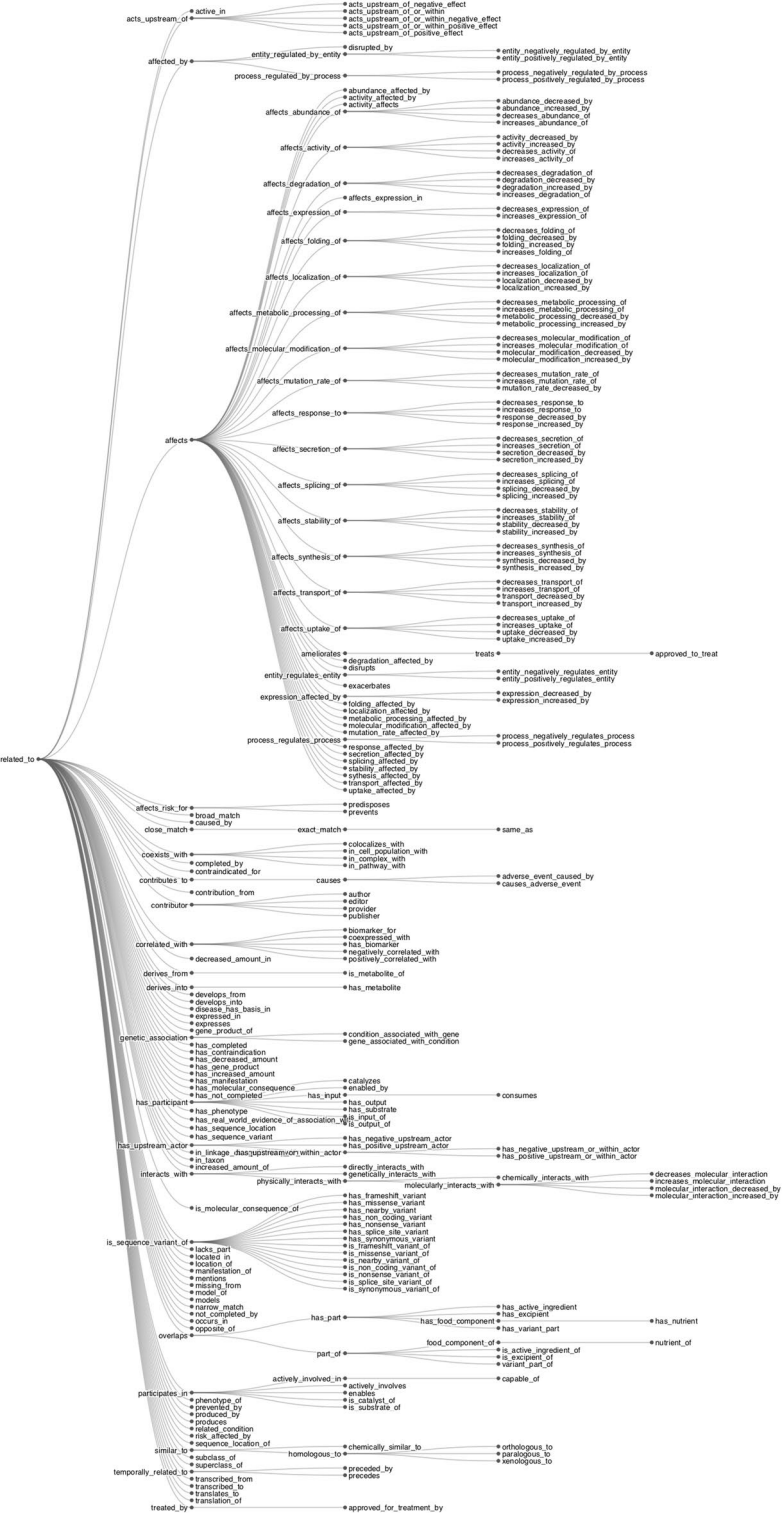
Edge predicate types in RTX-KG2.7.3 are based on the Biolink model version 2.1.0

The RTX-KG2.7.3 knowledge graph follows the Biolink model (version 2.1.0) for its semantic layer and (in RTX-KG2pre) its schema. RTX-KG2 uses Biolink’s category hierarchy for its concept (node) types (Fig. 2) and Biolink’s predicate hierarchy for its relationship (edge) types (Fig. 3). When mapping terms from their original sources to the Biolink terminology, the RTX-KG2 build system consults the Biolink model’s internal mappings in order to detect any inconsistencies between the two. Because relationship terms that are highly specific are often mapped to more generalized terminology, the original source’s phrasing is preserved in the relation property^2^. In addition to mapping upstream source relations to Biolink predicates, the RTX-KG2 build process coalesces edges that have the same end nodes and the same predicate (it does, however, preserve the provenance information from both of the coalesced edges).

The schema of the JSON serialization of RTX-KG2pre is documented in detail in the RTX-KG2 project area github:RTXteam/RTX-KG2 and summarized in the "Detailed schema for RTX-KG2" section. In brief, RTX-KG2 is serialized as a JSON object with keys nodes and edges, with the nodes object containing a list of serialized objects for the concept nodes in the graph, and with edges containing a list of serialized objects for the subject-object-relationship triples in the graph.

### Quality control and reproducibility

The RTX-KG2 build process incorporates multiple layers of quality control, including both automated and manual procedures (see the "Building RTX-KG2pre from upstream sources" section for details). As the first step in the build process, scripts validate the consistency of the RTX-KG2 semantic layer with the Biolink model. During knowledge integration, relationships whose subject or object nodes are not present in the knowledge graph are logged for offline investigation. A report of statistics on the RTX-KG2 knowledge graph is generated—including (i) node counts by knowledge source and by semantic type and (ii) edge counts by source and by relationship type—both before and after redundant edges are joined in the merge process. The procedure for RTX-KG2 builds includes a script-facilitated comparison of that report for the new build with the equivalent report for the previous build, in order to enable the build supervisor to recognize anomalously large (e.g., more than three-fold) changes in node or edge counts conditioned on source, category, or predicate. Once a new RTX-KG2c build is installed into the RTX-KG2 query API, functional correctness of the live API is verified using a Python-based test suite. Reproducibility of the RTX-KG2 build is enhanced by the intentional choice of using an ETL approach based on flat-file exports knowledge-source databases. To aid with versioning, most sources have their version stored in the name attribute in the node in RTX-KG2 that represents the source database.

### RTX-KG2 content and statistics

**Fig. 4:**
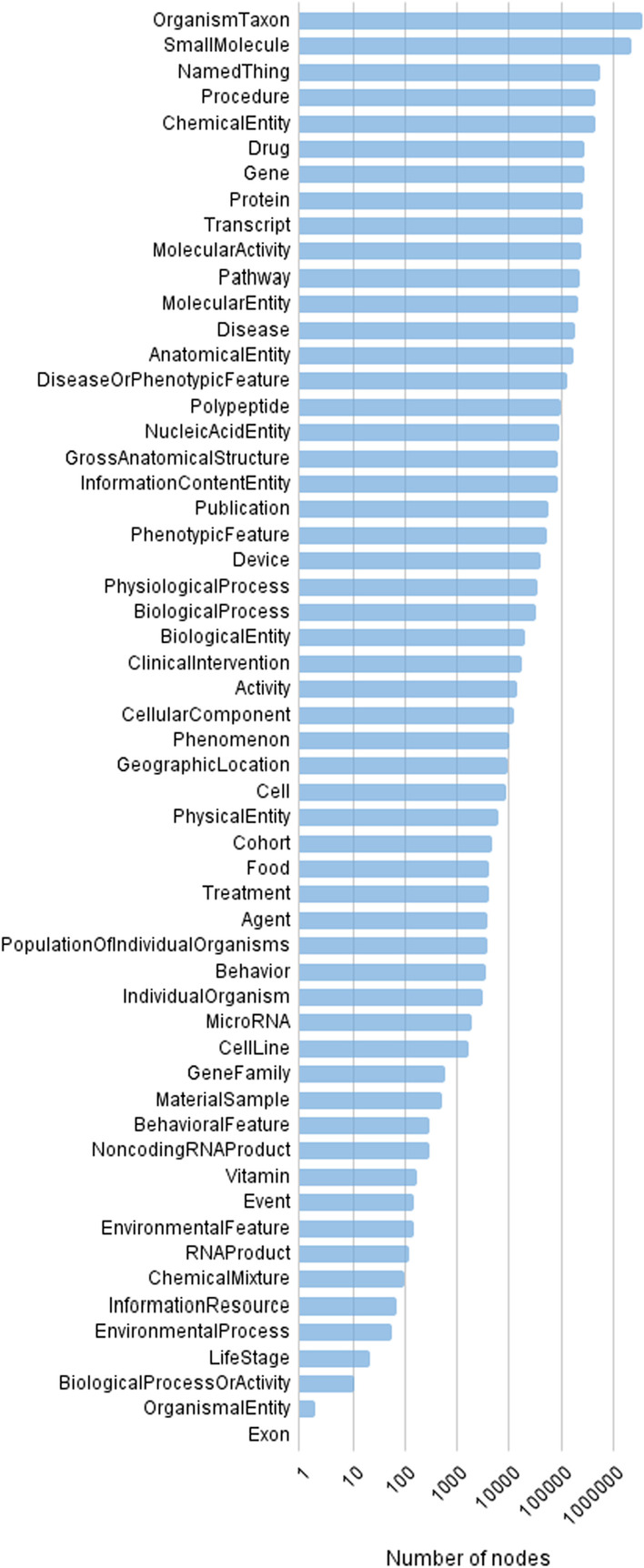
Number of nodes in RTX-KG2.7.3pre, by category

**Fig. 5:**
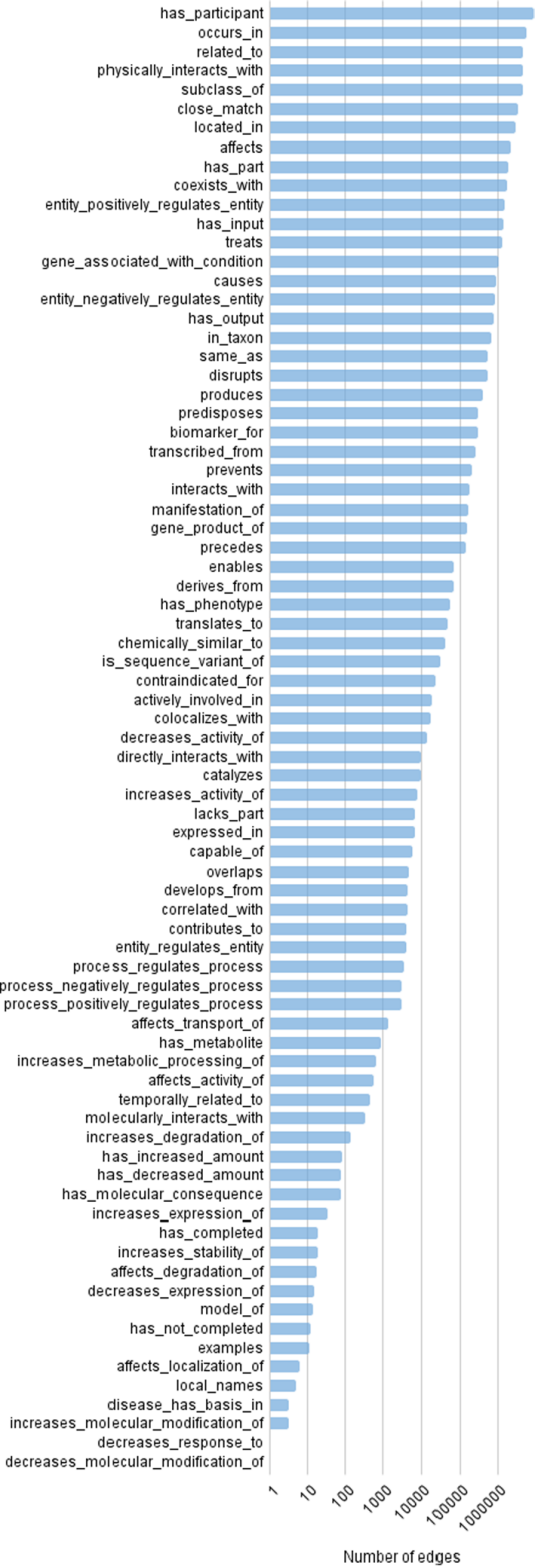
Number of edges in RTX-KG2.7.3pre, by predicate

The latest released version of RTX-KG2pre as of this writing, RTX-KG2.7.3, contains 10.2 million nodes and 54.0 million edges. Each edge is labeled with one of 77 distinct predicates (Biolink relationship types) and each node with one of 56 distinct categories (Biolink concept semantic types). In terms of frequency distribution, there is over six decades of variation across node categories (Fig. 4) and edge predicates (Fig. 5), with the dominant category being OrganismTaxon (reflecting the significant size of the NCBI organism classification ontology [103]) and the dominant predicate being has_participant (reflecting the significant size of the PathBank database [93]).

Fig.  5 shows a breakdown of edges in KG2.7.3 by their Biolink predicate.

KG2.7.3c contains 6.4 million nodes and 39.3 million edges, which is approximately 62% of the nodes and 73% of the edges of KG2.7.3pre, reflecting an expected reduction in node count due to canonicalization as well as due to post-canonicalization edge merging.

By number of edges, the largest contributing knowledge source for RTX-KG2.7.3pre is SemMedDB, which has 19.3M edges (comprising about a third of the edges in the graph), followed by PathWhiz (13.7M edges), NCBI Taxonomy (3.6M edges), and DrugBank (2.8M edges).

**Fig. 6:**
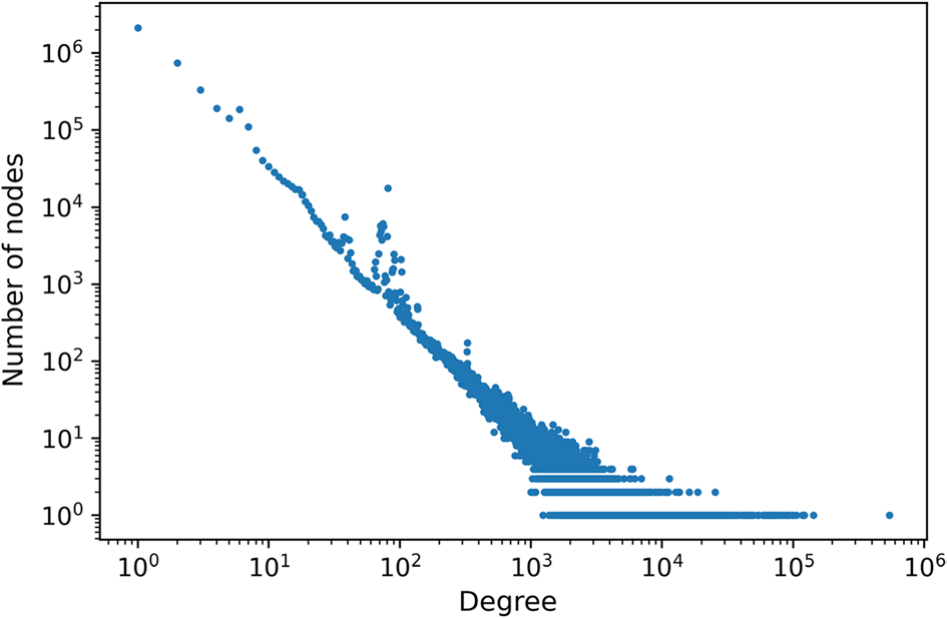
Node degree (in$+$out) distribution of RTX-KG2.7.3c

In terms of their total (i.e., in$+$out) vertex degree distributions, both KG2pre and KG2c appear to be approximately scale-free (Fig. 6) with a power law exponent of 2.43, meaning that the frequency of concepts with connectivity *k* decreases as ${\sim }k^{-2.43}$.

**Fig. 7:**
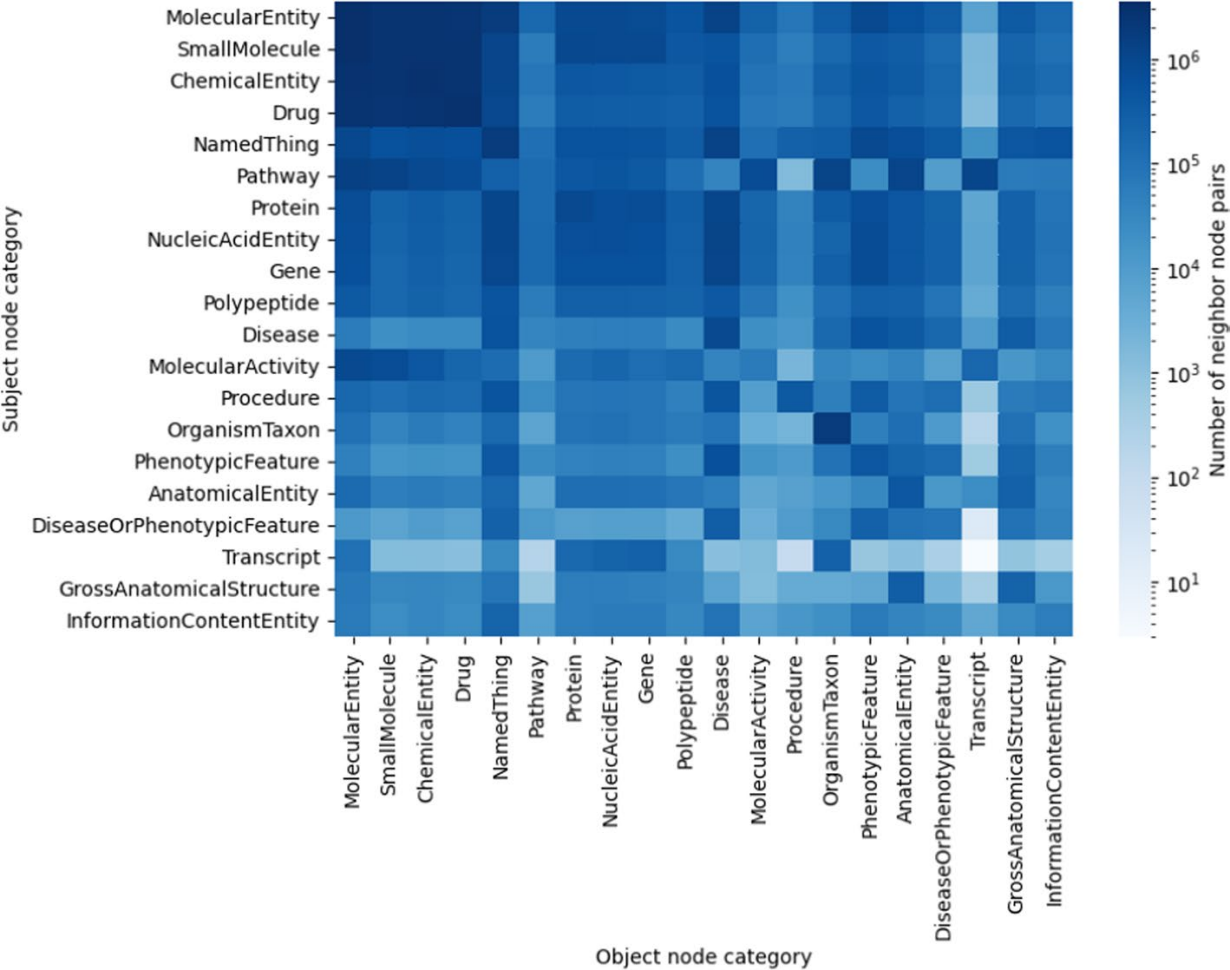
Node neighbor counts by category for the top 20 most common categories in RTX-KG2.7.3c. Each cell captures the number of distinct pairs of neighbors with the specified subject and object categories

Figure 7 highlights the frequencies of various combinations of subject node category and object node category appearing together in edges in KG2c, indicating (1) high levels of cross-category axioms among “molecular entity”, “small molecule”, and “chemical entity” and (2) high levels of connections between “pathway” and “molecular entity”, “small molecule”, “molecular activity”, “organism taxon”, “anatomical entity”, and “transcript”. Note that the category-category frequency heatmap is not expected to be symmetric for a knowledge graph (such as RTX-KG2) with a high proportion of relationship types that have non-reflexive subject-object semantics.

### RTX-KG2 access channels

The complete software code for building RTX-KG2 and for hosting an indexed RTX-KG2 graph database in Neo4j is publicly available in an open-source repository (see Availability of data and materials). In addition, the content of the latest RTX-KG2 graphs (version KG2.7.3) that we have built can be easily accessed via three different channels (see Availability of data and materials), depending on the use-case: (i) serialized flat-file download (as described below); (ii) REpresentational State Transfer (REST) [104] API (i.e., a web API); or (iii) web browser user interface, through the ARAX system. Tab-separated value (TSV) serializations of RTX-KG2pre and JSON serializations of RTX-KG2c are available in a public GitHub repository via the git-lfs file hosting mechanism, and their schemas are documented as described in the "RTX-KG2 schema and RTX-KG2pre Biolink compliance" section and in the RTX-KG2 documentation sections that are linked therein. RTX-KG2c can be queried via a REST API that implements the open-standard Translator Reasoner API (TRAPI) specification (github:NCATSTranslator/ReasonerAPI) and that is registered via the SmartAPI [64] framework and therefore discoverable using SmartAPI-associated tooling such as BioThings Explorer [46]. The RTX-KG2 API enables one-hop querying of the knowledge graph; queries are internally serviced by the PloverDB in-memory graph database (see the "Building RTX-KG2c, the canonicalized version of RTX-KG2" section). The ARAX API (arax.rtx.ai/api/arax/v1.2/openapi.json), which itself queries the RTX-KG2 API, can be used to achieve multi-hop RTX-KG2 queries. Further, RTX-KG2c is archived in Biolink Knowledge Graph eXchange [49] TSV format (KGX TSV format, documented at github:biolink/kgx) through the Translator Knowledge Graph Exchange (KGE; see Fig. 1) archive and registry system (github:NCATSTranslator/Knowledge_Graph_Exchange_Registry) (currently in testing phase).

## Utility and discussion

### Uptake and adoption

Due to its comprehensiveness and/or its speed, RTX-KG2 is already being used as a core knowledge provider (see github:NCATSTranslator/Translator-All/wiki/KG2) or knowledge graph by five diverse reasoning agents within the Translator system: ARAX [65], which our team developed and which provides sophisticated workflow capabilities and overlay of virtual edges for associations based on literature co-occurrence or network structural equivalence; mediKanren, which provides sophisticated network motif-finding and path-finding using the miniKanren logic programming language; BioThings Explorer, the engine for autonomous querying of distributed biomedical knowledge, described in the "Background" section; ARAGORN (github:ranking-agent/aragorn), a reasoning agent that has unique capabilities for coalescing and ranking knowledge subgraphs; and the Explanatory Agent (github:NCATSTranslator/Explanatory-Agent), a reasoning agent that uses natural language-understanding models in order to explain and rank results.

Use of the RTX-KG2 API appears to be increasing over time, with an average of 1,084 queries per day over the 9 months prior to this writing (September 2021 - June 2022) vs. an average of 1,417 queries per day over the last 3 months (April - June 2022), a 1.3-fold increase. Programs that query RTX-KG2 may optionally identify themselves; of the 51% of queries from the last 9 months in which the submitter was identified, approximately 63% were by the Explanatory Agent, 23% by ARAX, 7% by BioThings Explorer, and 7% by ARAGORN. Rather than using the RTX-KG2 API, the mediKanren reasoning agent uses a bulk download of RTX-KG2pre in conjunction with their own canonicalization algorithm.

In addition to its primary intended use-case for on-demand knowledge exploration and concept-specific reasoning, the RTX-KG2 knowledge graph can be used as a structure prior for data-driven network inference, for example, causal network learning. We have recently described a computational method, *Kg2Causal* [105], for using a general-purpose biomedical knowledge graph to extract a network structure prior distribution for data-driven causal network inference from multivariate observations. Using the predecessor graph, RTX-KG1 [59], we found that using a general knowledge graph as a prior significantly improved the accuracy of data-driven causal network inference compared to using any of several uninformative network structure priors [105]. To the extent that it incorporates multiple graph structural variations, RTX-KG2 can also be used as a test-bed for evaluating the performance of structurally generalizable graph analysis methods such as a subset of us have done for the case of a structurally generalizable node-node similarity measure [106].

Another application of RTX-KG2 is as training data for drug repurposing models; previous work by our team utilized RTX-KG2’s predecessor, RTX-KG1, to train a random forest model that predicts novel drug treatments for diseases [107]. More recent extensions to this work have utilized the canonicalized version of RTX-KG2 as training data.

### Comparison to other knowledge graphs/providers

To objectively evaluate the size and semantic richness of RTX-KG2 in comparison to other biomedical knowledge graphs, we compared it to four other knowledge graphs that are in active use for translational applications: Hetionet [36], SPOKE [37, 38], the SRI Reference Knowledge Graph [108], and ROBOKOP [44, 45]. For the counts of meta-triples (i.e., counts of edges with a given pattern of subject category, object category, and predicate, which provide quantitative information about the richness of the knowledge graph for providing relationships of particular types), we accessed the ROBOKOP and SPOKE graphs via their SmartAPI-registered Translator API (“TRAPI”) endpoints on March 15, 2022. For Hetionet and ROBOKOP, we used published node and edge counts [36, 45]. For the SRI Reference Knowledge Graph, we downloaded the graph in KGX TSV format from the Biomedical Data Translator Knowledge Graph Exchange (KGE) and analyzed the graph locally.

Node and edge counts for various knowledge graphs are shown in Table 2. The large number of edges in the ROBOKOP knowledge graph versus RTX-KG2 reflects the latter’s practice of joining edges that have identical subject node, object node, and predicate type. The large number of nodes in the SRI Reference Knowledge Graph [108] versus RTX-KG2pre is largely due to more InformationContentEntity nodes (3.7 million vs. 144,396) and SequenceVariant nodes (2.4 million vs. 0) in the former.

**Table 2:** Node and edge counts for various knowledge graphs

	Nodes	Edges
HETIONET, v1 [36]	47,031	2.3 million
SPOKE ver. 20190707 [38]	2.15 million	6.16 million
SRI Reference KG, ver. 2.0	20.2 million	41.6 million
ROBOKOP [45]	6 million	140 million
RTX-KG2.7.3pre	10.2 million	54.0 million
RTX-KG2.7.3c	6.4 million	39.3 million

Notably, RTX-KG2 contains a richer set of meta-triples (distinct combinations of subject node category, edge predicate, and object node category) versus the other knowledge graphs (Table 3); RTX-KG2c contains 4.6-fold more meta-triples than the second-ranked knowledge graph and 18.5-fold more meta-triples than the third-ranked knowledge graph, by meta-triple count. In general we would expect a graph with a greater number of meta-triples to be able to provide answers to a wider variety of queries, which is somewhat corroborated by the findings in the following paragraph. The finding that canonicalization increases the number of meta-triples can be understood as follows: since each node in RTX-KG2c has multiple categories, the number of meta-triples increases by the product of the count of subject node categories and the count of object node categories, for all subject-object node pairs joined by edges in the graph. The approximately 2.4-fold increase in the number of predicates in ROBOKOP versus in RTX-KG2 partially reflects the design choice in RTX-KG2 to standardize predicate directions and to orient triples so that for any inverted pair of predicates (e.g., “has part” and “part of”), only one of the predicates is used in the graph.

**Table 3:** Numbers of unique node categories, edge predicates, and meta-triples for various knowledge graphs

	Categories	Predicates	Meta-triples
SPOKE, TRAPI v1.2.0 API	14	24	44
SRI Reference KG, ver. 2.0	62	59	2047
ROBOKOP, TRAPI v1.2.0 API	20	185	2234
RTX-KG2.7.3pre	56	77	10,269
RTX-KG2.7.3c	56	77	41,225

Here, we have opted to compare RTX-KG2 to biomedical knowledge graphs that are canonicalized and still being updated (which excludes the pre-canonicalized Bio2RDF [41], which has not been updated since 2014).

**Fig. 8:**
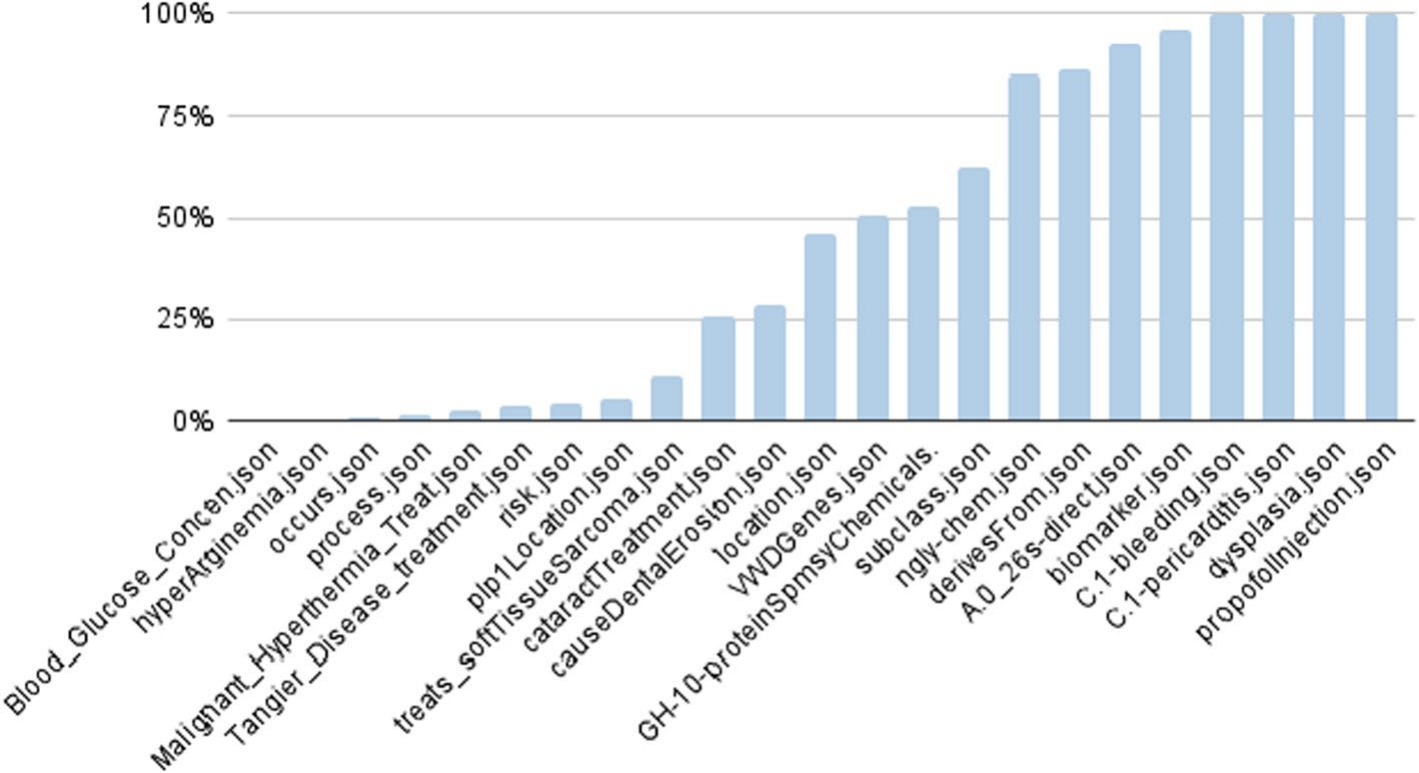
The proportion of results ARAX obtains for various one-hop queries when it is *not* allowed to use RTX-KG2 as one of its knowledge providers vs. when it is allowed to use RTX-KG2. A result of 100% means that RTX-KG2 provided no additional answers over ARAX’s other 12 Translator knowledge providers for that query; 0% means that all of ARAX’s results for that query came from RTX-KG2

To estimate the novelty of knowledge that RTX-KG2 provides over other knowledge providers, we ran a diverse set of one-hop queries^3^ through the ARAX reasoning agent and measured how many results were returned when ARAX was allowed vs. was not allowed to use RTX-KG2 as one of its knowledge providers. Omitting RTX-KG2 and relying on its 12 other Translator knowledge providers resulted in an average of 46% of the results compared to when RTX-KG2 was included. Figure 8 details the results for each query tested.

### Example use-cases

To help illustrate how to use RTX-KG2, an example RTX-KG2 API query is provided below. This query (which is written in TRAPI format) asks RTX-KG2 for genes related to Adams-Oliver syndrome:
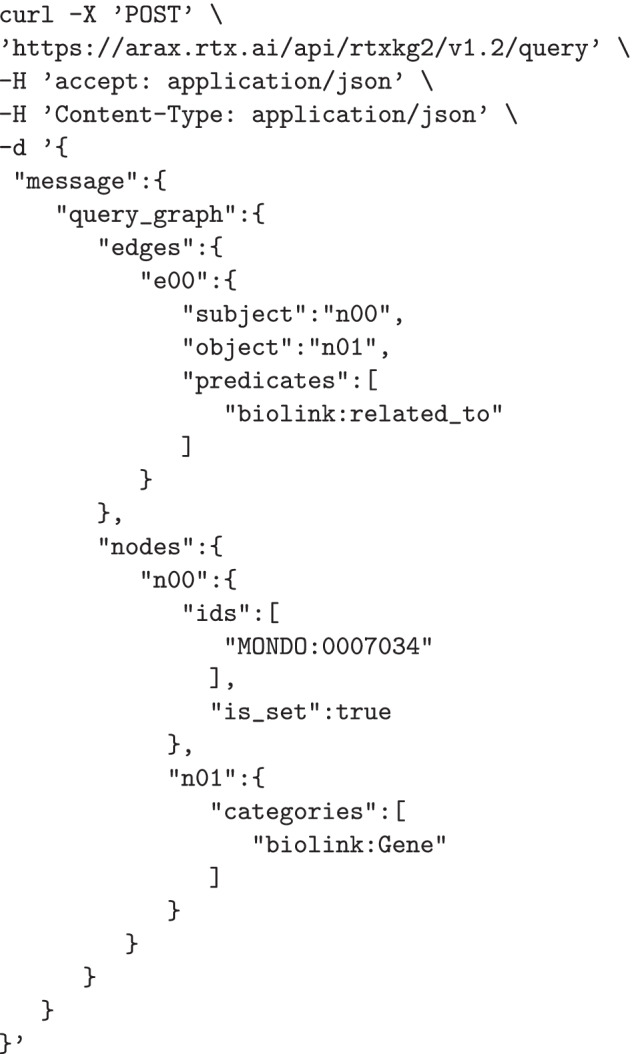


The response that RTX-KG2 returns to this query is also in TRAPI format and contains a ranked list of results and a knowledge_graph, per the TRAPI specification. The structure of each result object matches that of the query_graph, while the knowledge_graph contains all of the nodes and edges used in the results, decorated with evidence, provenance, and other information. As of June 30, 2022, this query returns 41 results, the top 10 of which are the genes *DOCK6*, *DLL4*, *NOTCH1*, *EOGT*, *RBPJ*, *ARHGAP31*, *CDC42*, *OGT*, *LFNG*, and *PAMR1*.

**Fig. 9:**
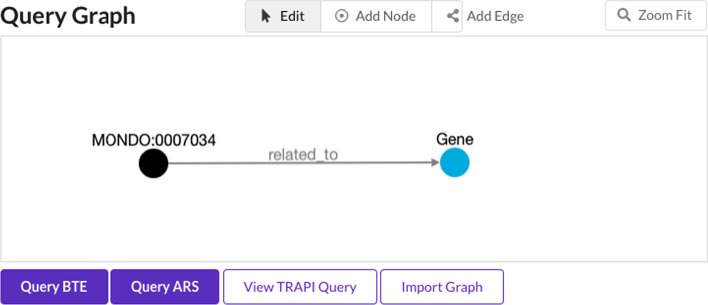
The BioThings Explorer query graph builder, which can be used to query RTX-KG2 among other Translator reasoning agents and knowledge providers

As stated in the "RTX-KG2 access channels" section, our ARAX reasoning system [65] provides a web browser interface (which is publicly available as described in Availability of data and materials) that can be used to both construct queries of RTX-KG2 and browse ranked results from those queries. The browser interface provides various capabilities including node synonymization; graphical layout and exploration of annotated result-graphs; and a graphical, point-and-click query builder. RTX-KG2pre serves as the data foundation for the ARAX Node Synonymizer (described in "ARAX Node Synonymizer" section), which is accessible via the ARAX UI (example: arax.rtx.ai/?term=naproxen) or programmatically via the ARAX API:
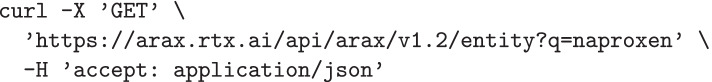


BioThings Explorer also provides a web browser-based user interface (biothings.io/explorer/query) including a query graph builder that can be used to query RTX-KG2 among other reasoning agents and knowledge providers. Figure 9 shows the example Adams-Oliver query in the BioThings Explorer query graph builder; click the “Query ARS” button to run the query and then the “Open ARS” button to go to a different user interface (provided by ARAX), in which users can select and explore result sets for different reasoning agents, some of which use RTX-KG2 as a knowledge provider (ARAX, BTE, ARAGORN, Explanatory Agent, and Unsecret Agent (which is based on mediKanren)).

### Discussion

In designing RTX-KG2, we chose five design principles that guided our selection of knowledge sources to incorporate as well as the architecture of the RTX-KG2 build system:

1. Source is publicly available in a flat-file (e.g., TSV, XML, JSON, DAT, or SQL dump) that can be downloaded via a script
2. Source is being maintained and updated periodically
3. Source provides knowledge triples that complement (i.e., not duplicate) what is already in RTX-KG2
4. Source connects concept identifier types that are already in RTX-KG2
5. Ideally, source provides knowledge based on human curation

Principle 1, and the deliberate choice of using an ETL approach, theoretically would allow RTX-KG2 to be reconstructed consistently and independently of the state of external APIs^4^. This is useful for reproducibility, since each knowledge source is stored in its original downloaded form as a build artifact. Using flat files instead of API interfaces also increases the probability that a future build can be completed successfully at any time, since it does not rely on multiple web services to be up for an extended period of time. Additionally, it is in many (though by no means all) cases computationally faster to ETL a file than to dynamically query an API over the Internet. With the ETL approach, for inter-ontology axioms, full interoperability is required and thus, generally full resource import (versus partial import as proposed previously [109]) is used. Development of RTX-KG2 is ongoing and our team welcomes recommendations of new sources to include, via issue reports on the RTX-KG2 GitHub project page (see Availability of data and materials).

In selecting the 70 sources for RTX-KG2, we generally adhered to the aforementioned principles but made a few exceptions based on specific trade-offs. For Principle 1, for one source (as described in the "Construction and Content" section), we used an API rather than a file download, and for the “via a script” part of Principle 1, we manually downloaded source dump files for DrugBank, UMLS, and SemMedDB (due to the download page requiring a login using a web browser) and RepoDB (due to its information on drug approval status). Some large databases such as SNOMED CT and MedDRA were not included in the UMLS ETL because they have additional restrictions under the UMLS Metathesaurus License (Appendix 2 and Sect. 12.3, respectively). For Principle 2, an exception was miRbase, due to the lack of a clear alternative source. For Principle 3, partial exceptions were made for the various pathway databases such as Reactome, PathWhiz/SMPDB, and KEGG, which have many overlapping pathways but which also had systems of pathway identifiers that needed to be included in RTX-KG2. Further, each of the pathway databases has different strengths: PathWhiz/SMPDB offer useful links to HMDB and DrugBank; Reactome is popular, trusted, and is well connected with sources like GO and CHEBI; and KEGG CURIEs are popular with users and link to CHEMBL, CHEBI, and GO. The primary exception to Principle 5 is SemMedDB which is based on natural-language processing of biomedical research article abstracts to extract knowledge triples. SemMedDB is particularly useful for downstream reasoning because of its breadth across biomedical literature and because it includes source article references for each triple. However, in the NLP arena, new methods such as REACH [21] and EIDOS [22] have been proposed that promise improved accuracy for determining event polarity (REACH) and detecting causal mechanisms (EIDOS); the potential of these methods to be applied to the full biomedical literature corpus remains to be fully explored.

It is a challenge to balance the importance of manually curated knowledge resources with those that provide numerical data and provenance (such as supporting publications) of their assertions. While these two are not mutually exclusive *per se*, relatively few knowledge sources seem to provide both. Increasingly, reasoning agents in the Translator system will use structured provenance and confidence information/annotations for edges in knowledge graphs such as RTX-KG2; the issue of knowledge sources that are important connectors in translational reasoning but do not provide structured provenance information is an ongoing problem [110]. A second notable challenge for computational biomedical reasoning is that of conflicting information between sources, which can occur due to data entry error at upstream sources, updating of concept identifiers or identifier prefixes, or changes in the semantic layer. In our experience, careful scrutiny of the build report (described in the "Quality control and reproducibility" section) is essential to catch systemic problems so that they can be addressed before the build is put into production. On the other hand, for ameliorating the effects of localized/incidental issues due to random errors in curated upstream sources (or incorrect triples called by the NLP algorithm used in SemMedDB), we have found that in computational reasoning, overlaying of multiple sources of quantitative evidence of association (such as co-occurrence in the literature or graph structural similarity) to be beneficial within the ARAX system [65]. Another source of potential errors is due to the absence of contextual information for a triple, for example, an interaction that applies only in a specific anatomic context; work is ongoing to extend the Biolink model and RTX-KG2 with qualifier semantics for annotating core triples with such contextual information, as described below.

Use of Biolink for the semantic layer for RTX-KG2 provides advantages both within and outside the Translator ecosystem. Normalization of the more than 1,228 source relationship types to a smaller set of predicates is necessary in order to produce a knowledge graph that is amenable to rule-based reasoning. The choice of 77 predicate types in Biolink is a trade-off, balancing simplicity of reasoning with semantic precision. Work on the Biolink model is ongoing to eliminate this trade-off by allowing for annotation of triples with statement qualifiers, subject qualifiers, object qualifiers, and typed associations. Further, substantial tooling within the Biolink project [51] has been developed to (i) provide ports of the Biolink model to six different open-standards representations (e.g., OWL, TTL, ShEx, JSON-schema, and GraphQL), (ii) provide a turn-key software package (KGX [111]) for import, export, and validation of Biolink-based knowledge graphs in various formats (e.g., TSV and JSON), (iii) provide a turn-key software library for programmatic manipulation of the Biolink model as Python or Java classes, and (iv) provide comprehensive cross-ontology mappings between Biolink and other high-level ontologies, for both entity types and predicates. Collectively, these efforts are expected to enable computational biomedical reasoning efforts that are outside Translator to interoperate at the semantic layer in order to integrate or query structured knowledge from RTX-KG2. The design of RTX-KG2 further prioritizes reproducibility by release-tagging, documenting versions of upstream sources (and the Biolink model version) with the RTX-KG2 release, including Biolink schema validation as a part of the release process, and having the build system environment setup automated and under source code control.

Our observation that RTX-KG2c has a scale-free degree distribution is consistent with previous reports from empirical studies of text-based semantic networks [112] and ontologies [113]. Further, the subgraph of RTX-KG2 that arises from the SemMedDB NLP analysis of PubMed/MEDLINE [114] and from the Gene Ontology [115] would be expected to follow a scale-free degree distribution, in accordance with Zipf’s Law. Quantitatively understanding the degree distribution of the knowledge graph (whether conditioned on the knowledge source, the relationship type, or the participating entity types) is relevant to quantitatively modeling the epistemic value of an edge in the graph (consider, for example, the downweighting by neighboring vertex out-degree in the PageRank algorithm [116]).

As described in the "Background" section, the Neo4j graph database management system is used by many biomedical knowledge graphs including Hetionet, Monarch, SPOKE, EpiGraphDB, and ROBOKOP. Using Neo4j to host RTX-KG2 has both benefits (specifically, the flexibility of the Cypher query language [117]) and drawbacks (in our testing, slow JSON loading performance and slow response times in comparison to an in-memory database). It was due to its drawbacks that we ultimately switched to hosting RTX-KG2c using a dedicated in-memory graph database engine, PloverDB (in the "Building RTX-KG2c, the canonicalized version of RTX-KG2" section), that we developed. On the other hand, our standard procedure of hosting a Neo4j database server for RTX-KG2pre has been invaluable as a debugging aid and for developing graph queries and analysis workflows.

**Fig. 10:**
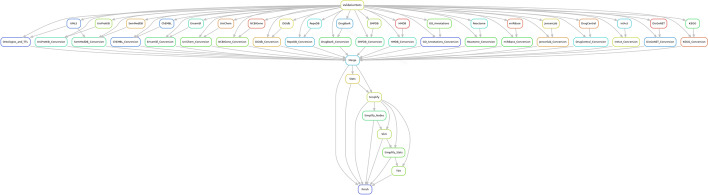
Flowchart of tasks for building RTX-KG2pre (the precursor stage of RTX-KG2) from 21 upstream knowledge-base distributions

The performance of the RTX-KG2 build process has been parallelized where feasible. Although ETL-focused, open-source workflow tools such as Pentaho Kettle and Apache Hop are available, we selected Snakemake because of its seamless integration with Python and its parallelization support. Since RTX-KG2 was intended to be a large knowledge graph, the build process was designed to limit the amount of time it would take to build, especially by using the ETL process described above and by utilizing a parallel build system. With parallelization, the RTX-KG2pre build takes about 47 hours versus $\sim$74 hours without parallelization. The system has the capability to carry out a partial rebuild (skipping the most time-consuming updates of UMLS and SemMedDB) which can reduce rebuild time by $\sim$60%. In addition, Snakemake functionality along with the ETL-based approach allows for unchanged source databases to be ignored until the Merge step (see Fig. 10), which reduces build time. While building RTX-KG2 requires short-term access to a system with at least 256 GiB of physical memory, once RTX-KG2 is built, it can be hosted on a commodity server system (e.g., 64 GiB of memory; see the "Requirements" section). New versions of RTX-KG2 are released at a rate of approximately once per month, with the median percentage increase in the number of edges per release being 0.8%, a relatively steady rate of growth consistent with the monthly rate of growth of abstracts in PubMed (0.6%).

## Conclusions

Despite the advances in the field outlined in the "Background" section, no open-source software toolkit was available that could integrate UMLS sources, SemMedDB, ChEMBL, DrugBank, SMPDB, Reactome, and 23 OBO Foundry ontologies (70 sources in all) into a single canonicalized knowledge graph based on the open-standard Biolink model as the semantic layer. To fill this gap and to provide a comprehensive knowledge-base to serve as as an efficient and accessible knowledge-substrate for a biomedical reasoning engine, we constructed RTX-KG2, comprising a set of ETL modules, an integration module, a REST API, and a parallel-capable build system that produces and hosts both pre-canonicalized (RTX-KG2pre) and canonicalized (RTX-KG2c) knowledge graphs for download and for querying. Quantitative usage information shows that RTX-KG2 is currently extensively used by multiple reasoning agents in the NCATS Biomedical Data Translator project, validating the ETL-focused, monolithic-graph, standards-based design philosophy that guided RTX-KG2’s development.

### Supplementary Information

**Additional file 1: Table S1.** Ontologies from the OBO Foundry that are included in RTX-KG2

## Data Availability

The code supporting the conclusions of this article is available in the GitHub repository github:RTXteam/RTX-KG2. Downloadable versions of RTX-KG2pre and RTX-KG2c are publicly available at github:ncats/translator-lfs-artifacts. The RTX-KG2 API is registered via the SmartAPI framework and can be reached at arax.rtx.ai/api/rtxkg2/v1.2/openapi.json. The web browser user interface for querying RTX-KG2 is publicly available at arax.rtx.ai/kg2.

## Appendix

### Technical details of database construction

#### Building RTX-KG2pre from upstream sources

The process by which the RTX-KG2 system builds its knowledge graph from its 70 sources—the first stage of which is diagrammed in Fig. 10)—begins by executing validation Python scripts (the “validationTests” task in Fig. 10) that ensure that the identifiers used in the RTX-KG2 semantic layer are syntactically and semantically correct within the Biolink model. Next, the build process executes in parallel the 21 direct-to-JSON and RDF-based ETL scripts (see second and third rows in Fig. 10 and in the "Sources and their file formats" section ) to produce a total of 21 JSON files (20 via the direct-to-JSON method and one via the RDF-based method) in the RTX-KG2 knowledge graph schema (described in the "RTX-KG2 schema and RTX-KG2pre Biolink compliance" section). Next, those JSON files are loaded and their object models are merged (via the “Merge” task) into a single self-contained graph that is then saved in an RTX-KG2-schema JSON file in which relationships consist of triples (subject, relation, object) where the relation is from any number of source-specific relationship vocabularies (totalling 1,228 source-specific relationship types in all). The RTX-KG2 object model is then simplified (via the “Simplify” task) by consolidating redundant relationships; a redundant relationship is where two or more sources assert the same triple (ibuprofen, treats, headache), in which case, the multiple relationships with identical triples are merged into a single relationship, with the multiple sources noted in the list-type provided-by relationship attribute (see the "RTX-KG2 schema and RTX-KG2pre Biolink compliance" section). Also in the Simplify task, relationship types are simplified by mapping each of them to one of 77 standardized relationship types (called “predicates”) in the Biolink model. This mapping process is controlled by a locally maintained, rule-based system encoded in Yet Another Markup Language (YAML) that is cross-checked by a validation script against the Biolink model at build time. In that ruleset, some semantic loss of precision is allowed, such as mapping DGIdb:partial_antagonist to biolink:decreases_activity_of, as a practical choice since the number of Biolink predicates (77) is much smaller than the number of total number of source relationship types. The Simplify task also maps source identifiers to Biolink “Information Resource” identifiers. In general, the Simplify task standardizes the graph with the Biolink model standards [49, 50]. We call the resulting graph *RTX-KG2pre* in order to emphasize that it is the precursor graph to the canonicalized RTX-KG2 graph (described below).

In the final step of the build process, the nodes and edges of RTX-KG2pre are imported into a Neo4j graph database (for details, see the "RTX-KG2 uses Snakemake for building RTX-KG2pre" section). During the RTX-KG2pre build, nodes (concepts) are assigned Biolink categories based on a rule-based system encoded in Yet Another Markup Language (YAML); at the start of each build, the YAML file is cross-checked by a validation script against known concept-to-category mappings in the Biolink model, to ensure correctness. The rule-based system allows for recursive assignment of a category to a class and all its subclasses, as well as to all concepts that have a specific CURIE prefix.

##### ARAX node synonymizer

As described in the "Building RTX-KG2c, the canonicalized version of RTX-KG2" section, in order to cluster concept identifiers into synonym groups (i.e., “canonicalize” the graph), the RTX-KG2 build system uses the ARAX [65] *Node Synonymizer* service, which takes into account four sources of evidence in the following order: (i) concept equivalence information obtained dynamically by querying a Translator web service API called the Standards and Reference Implementations (SRI) Node Normalizer (github:TranslatorSRI/NodeNormalization); (ii) biolink:same_as edges in RTX-KG2pre between RTX-KG2pre nodes; (iii) human-recognizable node (concept) name equivalence; and (iv) node semantic type compatibility. For dividing nodes into disjoint sets of equivalent nodes (i.e., Step 2 in the "Building RTX-KG2c, the canonicalized version of RTX-KG2" section), the Node Synonymizer goes through three passes of merging concepts in order to ensure that the partitioning of nodes is independent of the order in which the nodes are loaded into the Node Synonymizer. For choosing a canonical node for each concept cluster (i.e., Step 3 in the "Building RTX-KG2c, the canonicalized version of RTX-KG2" section), the Node Synonymizer uses a score-based system that flexibly enables incorporation of new heuristics. For the relatively small local table store for the ARAX Node Synonymizer, SQLite was chosen due to its combination of simplicity of deployment due to being serverless, its simplicity of use, and its faster response times vs. a network-based relational database management system solution. Performance of the Node Synonymizer is primarily tracked during the build process via a problems.tsv file, in which concept clusters that contain clashing categories (e.g., Drug and Disease) are recorded. The number of such “problem” clusters is monitored and any notable increases are manually debugged and corrected. While helpful, this performance metric relies on hard-coded definitions of which categories are considered clashing, does not catch potential merge errors that involve nodes of the same or compatible categories, and does not provide any insight into merge misses. In the future we plan to develop methods for more accurate performance assessment that do not have these limitations.

#### Detailed schema for RTX-KG2

RTX-KG2pre and RTX-KG2c are serialized as JSON with the former’s schema being largely a superset of the latter’s. The JSON-serialized RTX-KG2pre has top-level keys build, nodes, and edges, with the nodes object containing a list of serialized objects for the concept nodes in the graph, and with edges containing a list of serialized objects for the subject-object-relationship triples in the graph. Under the build key, a JSON object stores information about the version of RTX-KG2pre and timestamp of the build. Under the nodes key is a list containing a JSON object for each node. Each node object contains 16 keys corresponding to the node property types in RTX-KG2pre, detailed in Table 4. The id node property contains a compact representation of the canonical uniform resource identifier, i.e., a CURIE [118]. The category property of a node describes the node’s semantic type, such as biolink:Gene. Similarly, the edges key is a list containing a JSON object for each edge, with the edge JSON object containing the keys indicated in Table 5.

**Table 4:** Node properties in RTX-KG2pre and RTX-KG2c

	KG2pre	KG2c
all_categories		$\checkmark$
all_names		$\checkmark$
category	$\checkmark$	$\checkmark$
category_label	$\checkmark$	
creation_date	$\checkmark$	
deprecated	$\checkmark$	
description	$\checkmark$	$\checkmark$
equivalent_curies		$\checkmark$
full_name	$\checkmark$	
has_biological_sequence	$\checkmark$	
id	$\checkmark$	$\checkmark$
iri	$\checkmark$	$\checkmark$
knowledge_source	$\checkmark$	
name	$\checkmark$	$\checkmark$
provided_by	$\checkmark$	
publications	$\checkmark$	$\checkmark$
replaced_by	$\checkmark$	
synonym	$\checkmark$	
update_date	$\checkmark$	

The predicate edge property contains the relationship type from the Biolink relationship hierarchy (which is called a “predicate” in the Biolink model), and the relation edge property contains the source relationship, such as semmeddb:treats.

**Table 5:** Edge properties in RTX-KG2pre and RTX-KG2c

	KG2pre	KG2c
id	$\checkmark$	$\checkmark$
kg2_ids		$\checkmark$
knowledge_source	$\checkmark$	$\checkmark$
negated	$\checkmark$	
object	$\checkmark$	$\checkmark$
predicate	$\checkmark$	$\checkmark$
predicate_label	$\checkmark$	
provided_by	$\checkmark$	
publications	$\checkmark$	$\checkmark$
publications_info	$\checkmark$	$\checkmark$
relation	$\checkmark$	
relation_label	$\checkmark$	
subject	$\checkmark$	$\checkmark$
update_date	$\checkmark$	

The schema of the JSON serialization of RTX-KG2c is very close to that of RTX-KG2pre except that the former does not contain the top-level build key/object and, for each node object, RTX-KG2c contains some additional keys such as equivalent_curies, which enumerates the CURIEs of the nodes representing concepts that were semantically identified in the canonicalization step; all_names, which contains the name properties of the KG2pre nodes that were canonicalized together for the given KG2c node; and all_categories, which contains the category properties of the nodes that were canonicalized together for the given KG2c node. In addition, the ids of the corresponding KG2pre edges that each KG2c edge was created from are documented in the kg2_ids property.

#### RTX-KG2 build system and software

In this section we describe the Snakemake-based build system for RTX-KG2 and the build system infrastructure requirements for building the RTX-KG2pre and RTX-KG2c graphs.

##### Requirements

The software for building RTX-KG2pre is designed to run in the Ubuntu Linux version 18.04 operating system on a dedicated system with at least 256 GiB of memory, 1 TiB of disk space in the root file system, $\ge 1$ Gb/s networking, and at least 20 cores (we use an Amazon Web Services (AWS) Elastic Compute Cloud (EC2) instance of type r5a.8xlarge). The software for building RTX-KG2 makes use of AWS Simple Storage Service (S3) for network storage of both build artifacts and input knowledge source distribution files that cannot be retrieved by a scripted HTTP GET from their respective providers (see Table 6). These build files must be pre-staged in an AWS S3 bucket before the build process for RTX-KG2pre is started. For hosting RTX-KG2 in a Neo4j server, the system requirements are 64 GiB of system memory, 8 virtual CPUs, and $\sim$200 GiB of root filesystem storage (we use a r5a.2xlarge instance).

**Table 6:** Upstream source files that must be staged in S3 in order to build RTX-KG2

DrugBank	XML download	Requires browser to download
RepoDB	TSV download	Requires browser to download
SemMedDB	MySQL download	Requires browser to download
SMPDB PubMed IDs	CSV download	Obtained courtesy of Wishart Lab
UMLS metathesaurus	ZIP download	Requires browser to download

##### RTX-KG2 uses Snakemake for building RTX-KG2pre

RTX-KG2pre is built by a series of Python modules and bash scripts that extract, transform and load (ETL) 45 data downloads (corresponding to the rows of Table 1, with the “OBO Foundry” row counting for 21 separate downloads) from 24 source websites (see the "Sources and their file formats" section) into a standardized property knowledge graph format integrated with the Biolink model as the semantic layer. To maximize the reproducibility of RTX-KG2 builds, the build system is fully automated, including scripts for (i) setting up and configuring the build system to run, (ii) downloading and transforming data, and (iii) exporting the final graph to the graph database Neo4j. RTX-KG2 utilizes the Snakemake [63] workflow management tool to schedule multicore execution of the RTX-KG2pre build process. In addition to reducing the computational costs of the build and the amount of time it takes to run, Snakemake increases modularity by enabling individual components (and their upstream dependencies) to be executed, when necessary. This is particularly useful for allowing failed builds to resume at the point of failure (via a so-called “partial” build), once the root cause (which could be a parsing error from an upstream ontology, for example) has been fixed.

The build process starts with parallel source extractions, in which all of the source databases are downloaded and prepared for the format that their respective conversion script uses. Then, each upstream source’s dataset is processed by a Python conversion module. This converts each source’s data into the RTX-KG2pre JSON format (see the "RTX-KG2 schema and RTX-KG2pre Biolink compliance" section). Once all of the upstream data sources are converted into their RTX-KG2pre JSON file, a module merges all of them into a cohesive graph, such that no two nodes have the same CURIE ID. One of the challenges in this step is when different upstream sources provide different names for the same concept CURIE; the RTX-KG2pre build system addresses such name conflicts by having a defined order of precedence of upstream sources. After the merge step, edge source relation types (as described in the "Building RTX-KG2pre from upstream sources" section) are each mapped to one of 77 predicate types in the Biolink predicate hierarchy (see the "Building RTX-KG2pre from upstream sources" section), and redundant edges (same combination of subject node ID, object node ID, and Biolink predicate) are coalesced, with source relation information and source provenance information added to lists in the coalesced edge. The graph is then serialized as JSON (see the "RTX-KG2 schema and RTX-KG2pre Biolink compliance" section) and to TSV format. The build artifacts, including the unprocessed and processed JSON files and the TSV files, are uploaded into an AWS S3 bucket. RTX-KG2pre is then hosted in Neo4j on a smaller AWS instance (see the "Requirements" section); the Neo4j endpoint is mainly used for debugging.

##### umls2rdf and owltools

In the RTX-KG2pre build process, the 26 UMLS sources are ingested as TTL files that are generated in the extraction stage of the build process from the Rich Release Format (RRF [68]) UMLS distribution using two software programs, Metamorphosys [119] (to load the RRF files into the relational database system, MySQL) and umls2rdf [29] (to extract TTL files; see the "umls2rdf and owltools" section). Thus, a local MySQL database is used as an intermediate data source in the build process, from which TTL files are generated via umls2rdf.

**Table 7:** UMLS sources that are integrated into RTX-KG2.

UMLS semantic network	
Anatomical therapeutic chemical classification system	ATC
DrugBank database	DRUGBANK
Foundational model of anatomy	FMA
Gene ontology	GO
Healthcare common procedure coding system	HCPCS
Human gene nomenclature committee	HGNC
Health level seven version 3.0	HL7V3.0
Human phenotype ontology	HPO
ICD-10 procedure coding system	ICD10PCS
ICD-9, clinical modification	ICD9CM
Logical observation identif. Names & Codes	LNC
Medication reference terminology	MED-RT
MEDLINE plus	MEDLINEPLUS
Medical subject headings (MeSH) [2]	MSH
Metathesaurus	MTH
NCBI taxon	NCBI
National cancer institute thesaurus	NCI
National drug data file	NDDF
National drug data file-reference terminology	NDFRT
Online Mendelian inheritance in man [120]	OMIM
Physician data query	PDQ
Psychological index terms	PSY
RxNorm (normalized drug names)	RXNORM
National drug file	VANDF

See the "Discussion" section regarding UMLS sources that could not be included due to licensing

The build system uses the owltools software program (github:owlcollab/owltools) to convert biomedical ontologies (see Table 1 and Additional file 1: Table S1) in OWL format and the UMLS TTL files into OBO (Open Biological and Biomedical Ontology) JSON format for processing. The ontologies in OBO-JSON format are then loaded using the Python package ontobio (which, in turn, is based on the NetworkX graph library [121]) and processed/merged together, enabling use of cross-ontology axioms in determining concept semantic types.

## Abbreviations

ARAGORN: Autonomous Relay Agent for Generation of Ranked Networks
ARAX: Autonomous Relay Agent X
ARS: Autonomous Relay System
AWS: Amazon Web Services
BTE: BioThings Explorer
D2J: direct-to-JSON method
EC2: Elastic Compute Cloud
ETL: Extract-transform-load paradigm
GO: Gene Ontology
ICD: International Classification of Diseases
JSON: JavaScript Object Notation
KEGG: Kyoto Encyclopedia of Genes and Genomes
NCATS: National Center for Advancing Translational Sciences
NCBI: National Center for Biotechnology Information
OBO: Open Biomedical Ontologies
OWL: Web Ontology Language
RBM: RDF-based method
RDF: Resource Description Framework
REST: REpresentational State Transfer
RTX-KG2: Reasoning Tool X, Knowledge Graph Generation Two
RTX-KG2c: Reasoning Tool X, Knowledge Graph Generation Two, Canonicalized
RTX-KG2pre: Reasoning Tool X, Knowledge Graph Generation Two, Pre-canonicalization
S3: Simple Storage Service
SemMedDB: Semantic Medline Database
SMPDB: Small Molecule Pathway Database
SQL: Structured Query Language
Translator: NCATS Biomedical Data Translator
TRAPI: Translator Reasoner API
TSV: Tab-separated value
TTL: Terse RDF Triple Language
UMLS: Unified Medical Language System
XML: eXtensible Markup Language
YAML: Yet Another Markup Language

## Footnotes

1 An example identifier type prioritization would be for the semantic type “gene”, to prefer (from most to least preferred) identifier types from Ensembl Gene, NCBI Gene, and Human Gene Nomenclature Committee (HGNC).

2 This will be transitioning to the original_predicate property in the next release of RTX-KG2, for compatibility with recent changes in the Biolink standard.

3 github:NCATSTranslator/testing/ars-requests/not-none/1.2, accessed on June 23, 2022.

4 Note however, that one API is used in constructing RTX-KG2; see the "Sources and their file formats" section.
