# Supplementary material for: RTX-KG2: a system for building a semantically standardized knowledge graph for translational biomedicine
Source: BMC Bioinformatics. 2022 Sep 29;23:400. doi: 10.1186/s12859-022-04932-3 (PMC9520835; doi:10.1186/s12859-022-04932-3)
Supplement: Supplementary file 1 — Additional file 1: Table S1. Ontologies from the OBO Foundry that are included in RTX-KG2 [file 12859_2022_4932_MOESM1_ESM.pdf]

B Supplementary Material

Table S1: Ontologies from the OBO Foundry that are included in RTX-KG2.

Basic Formal Ontology	BFO
Chemical Entities of Biological Interest (ChEBI)	CHEBI [122]
Gene Ontology, with external relationships	`go-plus`
Relation Ontology	RO
Uberon multi-species anatomy ontology, extended with external relationships	UBERON
Foundational Model of Anatomy	FMA
Dictyostelium discoideum anatomy	DDANAT [123–127]
Cell Ontology	CL [128]
Food Ontology	FOODON
Human Developmental Anatomy, abstract	EHDAA2 [129]
Biological Spatial Ontology	BSPO
Human Phenotype Ontology	HPO
Neuro Behavior Ontology	NBO
NCBI organismal classification, taxslim subset	ncbitaxon [103]
Phenotype and Trait Ontology	PATO
Mondo Disease Ontology	MONDO
Disease Ontology	DO
Protein Ontology	PRO [130]
Interaction Network Ontology	INO
Genomic Epidemiology Ontology	GENEPIO
Molecular Interactions Controlled Vocabulary	MI
